# Illusory implications: incidental exposure to ideas can induce beliefs

**DOI:** 10.1098/rsos.240716

**Published:** 2025-01-22

**Authors:** Justin Mikell, Derek Powell

**Affiliations:** ^1^ University of California, Irvine, CA, USA; ^2^ Arizona State University, Glendale, AZ, USA

**Keywords:** illusory truth, metacognition, cognitive psychology, misinformation

## Abstract

Numerous psychological findings have shown that incidental exposure to ideas makes those ideas seem more true, a finding commonly referred to as the ‘illusory truth’ effect. Under many accounts of the illusory truth effect, initial exposure to a statement provides a metacognitive feeling of ‘fluency’ or familiarity that, upon subsequent exposure, leads people to infer that the statement is more likely to be true. However, genuine beliefs do not only affect truth judgements about individual statements, they also imply other beliefs and drive decision-making. Here, we consider whether exposure to ‘premise’ statements affects people’s truth ratings for novel ‘implied’ statements, a pattern of findings we call the ‘illusory implication’ effect. We argue these effects would constitute evidence for genuine belief change from incidental exposure and identify a handful of existing findings that offer preliminary support for this claim. Building upon these, we conduct three new preregistered experiments to further test this hypothesis, finding additional evidence that exposure to ‘premise’ statements affected participants’ truth ratings for novel ‘implied’ statements, including for considerably more distant implications than those previously explored. Our findings suggest that the effects of incidental exposure reach further than previously thought, with potentially consequential implications for concerns around mis- and dis-information.

## 1. Introduction

Every day, people are faced with a barrage of unsupported claims, from pestering ad campaigns to blatant disinformation on social media. Altogether, we live within an information environment that is more connected and more saturated than ever before in human history [1]. If we are sufficiently critical consumers of media, can we benefit from this rich access to information without being exploited by advertisers or misled by misinformation? Perhaps not. Numerous psychological findings indicate that incidental exposure to ideas makes those ideas appear more true, a finding commonly referred to as the ‘illusory truth’ effect [2–6]. This effect suggests that we cannot exist in an environment of mis- and dis-information without being affected by it—without exposure to these ideas distorting our sense of what is true.

Studies examining the illusory truth effect typically proceed in at least two phases. At exposure, participants are introduced to a set of false statements. Typically, the statements are part of a true/false quiz, or a cover story explains they are part of some other innocuous judgement task. Then, after some intervening time ranging from minutes to weeks, participants are asked to judge whether these statements are true. On average, participants rate the statements as more ‘true’ when they have been exposed to them previously—an illusory truth effect.

Decades of research have demonstrated the consistency and robustness of the illusory truth effect (e.g. [2]). The effect has been demonstrated for frivolous trivia questions (e.g. [4,7–9]) as well as consequential fake news headlines [5]. The illusory truth effect has also been shown to be robust across people with different levels of cognitive ability, need for cognitive closure and cognitive styles [6].

The effect is one of ‘illusory’ truth because it occurs following ‘incidental exposure’ to statements. That is, it occurs when the statements are seen or heard in a non-communicative context, such as when they are read during a true/false quiz. Generally, being told something—even by a source of unknown trustworthiness—is a prima facie reason for believing it (10; also see [11]). But reading a statement on a true/false quiz is not a valid reason for believing it; the statement is just as likely to be false as to be true.

### 1.1. Theories of the illusory truth effect

Why should incidental exposure affect later ratings of truth? Gilbert [12] proposed that any idea entertained is by default first believed and must later be ‘unbelieved’. This perspective offers one explanation for the illusory truth effect: people might simply believe what they are told, even when that exposure is incidental or the pragmatics of the situation indicates that they should withhold judgement [13–15].

In contrast, rather than imagining people are truly so credulous, another set of theoretical accounts attributes illusory truth effects to metacognitive experiences of fluency or familiarity [2], rather than genuine influences on beliefs. Research on metacognition has shown that people’s behaviour and judgements are not only influenced by the content of their thoughts but also by the phenomenological experience of processing those thoughts [16,17]. Metacognitive processing ‘fluency’ is the experienced ease of processing or thinking about information [16,18]. When thinking is easy and fluent, people tend to judge the targets of that thinking more favourably than if thinking is challenging and disfluent [19]. A number of findings have shown that processing fluency contributes to the illusory truth effect: incidental prior exposure to statements makes later processing of those statements more fluent, and this metacognitive experience of fluency leads people to rate those statements as more true [3,8,9,20].

Metacognitive fluency can derive from different sources and several different types have been shown to influence ratings of truth. Fluency can be *perceptual*: statements printed more clearly or in easier-to-read fonts are rated as more true [21]. Fluency can be influenced by *memory retrieval*, such that statements that are easier to retrieve from memory are rated as more true [22]. And fluency can also be semantic or *conceptual* in nature: concepts that are semantically primed are seen as better answers to trivia questions [23], and statements evoking previously activated concepts are rated as more true [14].

Other researchers have attributed the illusory truth effect to familiarity (e.g. [24,25]). Prior exposure to statements engenders a feeling of familiarity upon re-exposure to those statements. This metacognitive feeling of familiarity provides a cue that leads people to infer that the statement is more likely to be true.

Both metacognitive accounts provide a similar overall view of the illusory truth effect: roughly, when asked whether something is true or false, people search their memory for knowledge pertaining to their belief in the statement, but they also rely on their metacognitive experiences of familiarity and fluency. Reliance on metacognitive experiences may be, at least to some extent, adaptive: familiarity and fluency are both signs that we have ‘heard this somewhere before’. According to foundational theories of communication, a general assumption that communicators strive to make true utterances is a prerequisite for successful communication and social functioning [10]. Thus, if having ‘heard something before’ suggests that someone said it, then ecologically this is a reasonable cue to the truth. On this account, psychological studies that elicit the illusory truth effect hijack these metacognitive heuristics: they provide this sense of familiarity or fluency, but from a communicative context that lacks any reasonable assumption of truth.

### 1.2. Incidental exposure and belief

There is something unsettling about the illusory truth effect and the lack of agency it seems to imply about our own thinking. Even more worrisome, there could be important societal implications if merely being exposed to an idea causes people to adopt it as a belief: Pennycook and colleagues [5] argue that the illusory truth effect, combined with an environment of pervasive misinformation, has important consequences for the functioning of democratic society (also see [26]). The illusory truth effect suggests that incidental exposure to misinformation could have impacts that cannot be stopped by fact-checking labels [5], nor effectively curbed by retractions or corrective information (cf. [27,28]).

The extent of these impacts depends on the source of the illusory truth effect, whether it is a product of metacognitive processes or results from the adoption of incidentally experienced information as beliefs. Beliefs inform actions and decision-making: when truly believed, misinformation can have serious consequences. For instance, a recent study found that people who marked just one piece of vaccine misinformation as accurate were nearly twice as likely to be unvaccinated against COVID-19 compared with those who did not endorse any vaccine misinformation [29]. In addition, beliefs imply other beliefs: for instance, believing that ‘it is sunny out’ implies the belief that ‘it is daytime’. And believing misinformation, such as ‘Dominion voting machines were rigged in the 2020 U.S. election’ might imply the belief that ‘Donald Trump actually won the 2020 U.S. election’.

Some researchers have argued that the illusory truth effect itself demonstrates an influence of prior exposure on beliefs (e.g. [13]). Yet as these points illustrate, there is more to *belief* than ratings of truth. Though a person’s assent to a proposition (whether they agree with or judge statements expressing it to be truthful) is an important marker and convenient measure of belief, these other features are just as essential. Moreover, we argue that where the effects of incidental exposure on truth judgements are plausibly attributable to well-established metacognitive processes, these effects do not generally constitute evidence for a change in beliefs.

**Figure 1.**
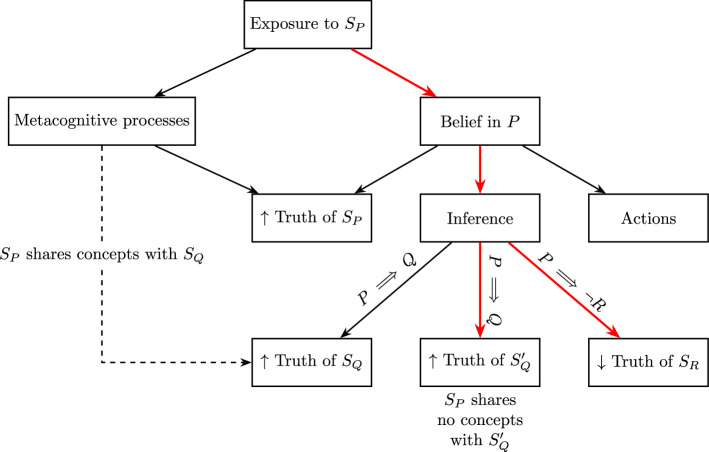
Diagram depicting the theorized causal relations between latent beliefs, metacognitive experiences and truth ratings for different types of statements. We highlight the paths from observable behaviour that can only be traced back to exposure through beliefs in red.

Nevertheless, these wider consequences can help to determine whether incidental exposure to information modifies beliefs or influences judgements solely through metacognitive processes. Figure 1 depicts a set of hypotheses concerning the causal relationships between latent beliefs, metacognitive experiences and overt behaviours such as truth ratings and actions. As shown in the figure, experiences or manipulations that affect metacognitive experiences, such as incidental exposure, might produce increased truth ratings for statements expressing P (either through lexical or conceptual similarity). However, beliefs should be expected to produce additional consequences. Here, we focus on inferences: a manipulation affecting belief in P should increase truth ratings for statements expressing some proposition Q where P implies Q, and decrease belief in R where P implies ¬R. Figure 1 also depicts a possible relation between metacognitive experiences and statements expressing Q. Where a statement $S_{Q}$ expressing Q invokes a similar set of concepts as $S_{P}$, conceptual fluency might produce increased truth ratings.

Crucially, our diagram draws no connection between metacognitive experiences and reduced truth judgements for statements expressing R where P implies ¬R. Such a connection would require a cogent theory of how conceptual ‘disfluency’ might be engendered to reduce truth judgements without the process of inference that would implicate changes in belief. Current accounts have not developed such a theory (e.g. [16,30]), and it appears unlikely to be workable: people frequently encounter chains of statements invoking different concepts, yet they do so seemingly without issue, as considering one topic does not necessarily make it more difficult to consider another unrelated topic.

Applying the causal model sketched in figure 1, cases where incidental exposure to a proposition P increases truth ratings for statements $S_{P}$ expressing that proposition do not, in our view, constitute evidence that incidental exposure has affected beliefs, since there is a potential causal pathway between exposure and this result that does not pass through belief. Likewise, evidence of exposure to $S_{P}$ increasing truth ratings for $S_{Q}$, where $S_{Q}$ shares a similar set of concepts with $S_{P}$, does not constitute evidence for effects on beliefs. However, evidence that exposure decreases truth ratings for $S_{R}$ or increases truth ratings for *S*′*_Q_*, where *S*′*_Q_* does not directly share concepts with $S_{P}$, would constitute evidence for effects on beliefs.

Practically speaking, the extent to which the effects of incidental exposure operate entirely through metacognitive processes stands to determine the extent of their real-world consequences. Generally, it is the wider consequences of belief (beyond truth ratings for a specific statement) that should be most concerning. If effects of incidental exposure operate only through metacognitive influences, then the impact of prior exposure depends on a subsequent re-exposure to a now fluent or familiar situation or statement (which might include one activating similar concepts). To be sure, metacognitive effects might have important consequences even if they do not affect beliefs, such as increasing the tendency to share information online after having been exposed previously [31]. Still, the impact of metacognitive effects seems less likely to drive inferences or decision-making in more novel situations. Instead, their influences would be constrained to operating within contexts similar to the initial exposure.

### 1.3. Is there an ‘illusory implication’ effect?

A crucial feature of belief is that beliefs are connected with other related beliefs. Thus, one way to test whether incidental exposure affects beliefs is to examine whether incidental exposure to statements can affect truth ratings for other related or implied statements in ways that cannot be attributed to metacognitive effects. We refer to this hypothetical impact of incidental exposure on implied statements as the ‘illusory implication’ effect.

A key test of the illusory implication effect as opposed to other illusory truth effects is whether prior incidental exposure can *reduce* judgements of truth. The experience of fluency is inherently relative: a metacognitive experience is only fluent or disfluent with respect to some point of comparison. For instance, utterances can be made relatively fluent by rhyming [32] or they can be made relatively disfluent with the addition of speech markers such as ‘um’ and ‘uh’ [33]. However, in the case of repetition-induced fluency (whether conceptual, lexical or otherwise), repetition can produce fluency, but it is less clear how such a manipulation might produce disfluency. Thus, it is difficult to see how the effects of incidental exposure that reduce truth ratings can be explained by metacognitive factors.

A handful of existing findings provide some preliminary evidence for our hypothesized illusory implication effect. A number of researchers have found that incidental exposure affects truth ratings for novel statements contradicting previously seen statements [13–15]. To our knowledge, three studies have found evidence for this specific pattern of influence: Garcia-Marques *et al.* [13] (*n* = 58), Silva *et al.* [14] (exp. 2, n=45) and Unkelbach & Rom [15] (exp. 3; n=89). Such a pattern is not easily attributable to purely metacognitive processes.

Garcia-Marques and colleagues [13] induced incidental exposure by asking participants to rate how interesting statements were, warning them that half were true and half false. After this initial task inducing incidental exposure, a subsequent quiz featured a mix of novel statements, repeated statements and statements contradicting those previously seen either immediately or after a one-week delay. In the immediate test condition, contradictory statements were rated as less true than novel statements.^1^

Silva and colleagues [14] found that exposure to statements increased truth ratings for paraphrases of those statements that expressed the same proposition while sharing only minimal surface-level linguistic features. These researchers explained their findings as resulting from conceptual fluency produced by the initial exposure to statements: prior exposure ‘activates’ the relevant concepts, easing their retrieval and use in processing subsequent statements and thereby producing different metacognitive experiences. However, Silva and colleagues [14] also found that exposure to statements *decreased* truth ratings for rephrased contradictions of those statements, which we identify as a key test of an ‘illusory implication’ effect.

Similarly, Unkelbach & Rom [15] examined both implied and contradictory statements. Consistent with the hypothesized illusory implication effect, they found that exposure to statements increased truth ratings for novel implied statements (exp. 4) and that statements saw a significant decrease in truth ratings following incidental exposure to their contradictions (exp. 3). Crucially, the results of this experiment appear to preclude any purely fluency-based account of reduced truth ratings following exposure to contradictory statements: these researchers found that responses were faster for contradictions of previously seen statements than for novel statements, indicating that processing of these statements was *more* rather than less fluent. Thus, even if an appropriate notion of ‘conceptual disfluency’ could be developed, it is unlikely it could be an appropriate account of these findings [15]. Instead, they and other authors theorize that people tend to believe statements so long as they are generally coherent with respect to existing knowledge, even if they are presented in a context of incidental exposure [12–15].

However, these last two sets of findings must be qualified by the exposure context used in these studies: these researchers exposed participants to statements by asking them to make innocuous judgements about these statements (e.g. ‘how interesting is this statement?’) but did not warn them prior to this exposure that the statements could be true or false [14,15]. The pragmatics of this task is therefore ambiguous: participants might have imagined they were being asked to rate the interestingness of a set of entirely true statements and might have then believed those statements for that reason. In the absence of strong and clear task pragmatics (e.g. a true-or-false quiz), explicit warnings are key to establishing incidental exposure as we have defined it here.

The presently hypothesized illusory inference effect is a key theoretical link demonstrating the connection between incidental exposure and *beliefs*, and all of the attendant downstream consequences associated with beliefs. A handful of prior findings suggest the existence of such an effect, yet we do not take this existing evidence to be dispositive.

### 1.4. The present studies

Existing research examining the effects of incidental exposure on implied statements has two key limitations. First, it has often been conducted in ambiguous contexts where participants might not understand that statements might be true or false and that therefore do not always clearly induce incidental exposure, as we have defined it here. We address this in our study designs, by paying careful attention to the pragmatics of the exposure tasks to ensure they constitute clear cases of incidental exposure. Second, prior work has primarily examined effects on direct contradictions of originally seen statements (or paraphrases of direct contradictions) (but see [15]). Here, we sought to further test for an ‘illusory implication effect’ by testing the effect of incidental exposure to statements on their more distant logical implications. To do so, we designed a series of studies to examine whether incidental exposure to one statement (the ‘premise’) could affect truth ratings for another statement this premise would logically entail being either true or false (the ‘implication’).

In our studies, we sought to eliminate as much as possible the role of perceptual and conceptual fluency in affecting truth judgements. These efforts are aimed at demonstrating as clearly as possible a causal pathway between incidental exposure and effects on truth ratings through changes in belief. That is, establishing a pathway from exposure to increased truth ratings for implications Q and decreased truth ratings for contradictions R. Compared with prior work, we developed materials involving more distant inferences that do not afford direct one-to-one mappings between shared concepts in the premise and implication statements. Thus, any reduction in truth ratings for the contradicted statements following exposure to the premise statements are not reasonably attributable to disfluency (closing the alternative pathway depicted in figure 1). For instance, one statement pair was the premise statement, ‘No U.S. astronauts have died since the Challenger explosion in 1986’ paired with the implied statement, ‘The space shuttle Columbia disintegrated over Texas in 2003’ (true, but contradicted by the ‘premise’). In contrast with prior work examining conflicting statements, there is no direct one-to-one mapping of concepts between the two statements. Instead, a chain of inferences must be developed for a conclusion to be drawn: that the space shuttle Columbia contained astronauts, that its disintegration would have killed them, and that 2003 is later than 1986.

Additionally, we make several further contributions towards definitively demonstrating the illusory implication effect beyond existing studies by (i) testing the effect with large samples of participants (present studies N=1020), (ii) utilizing modern hierarchical statistical modelling methods to generalize beyond the exact items used, and (iii) taking great pains to ensure that participants’ truth ratings are not influenced by task demands (experiments 2 and 3). Across our studies, we find further compelling evidence for this ‘illusory implication’ effect, indicating that incidental exposure has genuine impacts on beliefs extending beyond those attributable to metacognitive cues.

## 2. Transparency and openness

All experiments were preregistered prior to data collection and all materials, data and analysis code have been made publicly available on the Open Science Foundation website (https://osf.io/znq3y/). In these preregistrations, we report our approach to determining sample sizes, all data exclusions, all manipulations and all measures in each study. The analytic plan for experiments 1 and 2 was preregistered in terms of its general designs and a more detailed analytic plan was preregistered for experiment 3. Where appropriate, we explicitly differentiate between preregistered analyses and non-preregistered analyses in our results. All statistical models were fit using the brms R package [35]. Our study was reviewed and approved by the Arizona State University Institutional Review Board under project identifier STUDY000122. Participants provided informed consent before beginning the study.

## 3. Experiment 1

### 3.1. Methods

#### 3.1.1. Participants

A total of 400 participants were recruited through CloudResearch. Basic attention check questions were used to screen inattentive participants. These questions asked participants to simply give a particular response. Participants who failed any attention checks or who indicated during debriefing that they had searched online for question answers were excluded from the analysis. This left a final sample of 378 (157 female, median age 36 years old).

#### 3.1.2. Materials

There were 24 pairs of statements presenting claims about history. Each pair consisted of a ‘premise’ statement and an ‘implication’ statement. Each ‘premise’ statement was a falsehood that either entailed or contradicted the ‘implication’ statement. Of the pairs, 12 had premise statements implying that a true statement was false (‘contradicted’) and the other 12 had premise statements implying another false statement was true (‘entailed’). For instance, one ‘contradicted’ pair was the premise statement, ‘No U.S. astronauts have died since the Challenger explosion in 1986’ paired with the implied statement, ‘The space shuttle Columbia disintegrated over Texas in 2003’ (true, but contradicted by the ‘premise’). An example of an ‘entailed’ pair was the premise statement, ‘The Tour de France has been held every year since its inception in 1903’ paired with the implied statement, ‘The Tour de France was still held during WWI and WWII’ (false, but entailed by the ‘premise’).

#### 3.1.3. Procedures

The study proceeded in three phases: (i) an exposure phase, (ii) a distraction phase, and (iii) a final testing phase.

At the beginning of the study, participants were randomly assigned to either the ‘fact’ (*n* = 100) or ‘quiz’ (*n* = 300) exposure condition and to one of three counterbalancing conditions. At the exposure phase, participants were presented with a subset of the ‘premise’ statements (8 of the 24 total, counterbalanced across participants) as well as a set of control statements (12 true, 4 false). Participants assigned to the quiz and fact conditions received different instructions and performed different tasks in the exposure phase.

Participants in the fact condition were told the study’s main purpose was to learn more about how people learn and apply new knowledge. They were informed that the initial set of statements they would see were all facts and were asked to rate how surprising each ‘fact’ was to them. The true purpose of this condition was to test whether participants would successfully draw inferences from the ‘premise’ to the ‘implied’ statements when told that the premises were true.

Participants in the quiz condition were told that they were to be given a true/false quiz, so that some of the statements would be true and some false. These participants rated how confident they were that each of the presented statements was true or false. The purpose of the quiz condition was to provide ‘incidental exposure’ to these statements and to test for illusory truth and illusory implication effects.

After their initial exposure to the statements, participants provided basic demographic information and were then presented with the expanded seven-question version of the cognitive reflection task (CRT [36,37]). These tasks served to provide a period of distraction between exposure and test.

Finally, the testing phase for all participants consisted of a true-or-false quiz, where participants were asked to rate their confidence that each statement was true or false. These ratings were made on a six-point scale from ‘definitely false’ to ‘definitely true’. The items tested included both ‘premise’ and ‘implied’ statements from the 24 item pairs. Participants were quizzed on the eight previously exposed premise statements, as well as their corresponding ‘implied’ statements. For comparison within subjects, they were also quizzed on eight new premise statements and eight unrelated implication statements. Participants were randomly assigned into three counterbalancing conditions that varied which of the statement pairs were assigned to each exposure/test combination.

We tested for the illusory truth effect by comparing truth ratings for premise statements seen during exposure to those that had not been seen. Similarly, we tested for the illusory implication effect by comparing truth ratings for implication statements whose corresponding premise statements were and were not presented during the exposure phase. If incidental exposure to ‘premise’ statements affects participants’ judgements of the ‘implication’ statements, this would be evidence for an illusory implication effect.

At the end of the study, participants were debriefed and presented with a list of the false statements they had seen.

### 3.2. Results

Participants made their truth ratings in the test phase on a six-point scale from ‘definitely false’ to ‘definitely true’. To properly treat these Likert-style responses, the truth ratings were analysed using multi-level Bayesian cumulative ordinal regression models [38] with random intercepts and slopes for participants and items. Cumulative ordinal regression models assume that participants’ discrete responses are driven by a continuous latent variable and a set of k-1 thresholds determining the range of the continuous variable corresponding to each of the ordinal response options. This model helps to account for potential differences in scale usage as well as the bounded nature of the response scale.

All models were fit using the brms R package [35], with model posteriors estimated using the No-U-Turn Markov chain Monte Carlo (MCMC) sampler implemented in Stan. Four MCMC chains were run for each model, with 2000 samples (1000 burn-in) drawn from each. MCMC diagnostics indicate good convergence: chains were assessed for convergence with $\hat{R}$ (all parameters $\hat{R}=1\pm 0.01$) and the total estimated effective sample size was verified to be greater than 1000 for all parameters [39]. All models were fit without divergences and without warnings for the estimated Bayesian fraction of missing information (eBFMI).

#### 3.2.1. Fact condition

First, we examined truth ratings for the premise and implication statements among participants who were told that the false statements were true at exposure (‘fact’ condition). As expected, exposure to the premise statements when presented as ‘facts’ increased endorsement at test, β= 1.191, 95% CI [0.996, 1.384]. In addition, participants successfully drew inferences from these ‘facts’, resulting in a decreased accuracy for the implication statements at test, β= −0.347, 95% CI [−0.599, −0.096].

#### 3.2.2. Quiz condition

**Figure 2.**
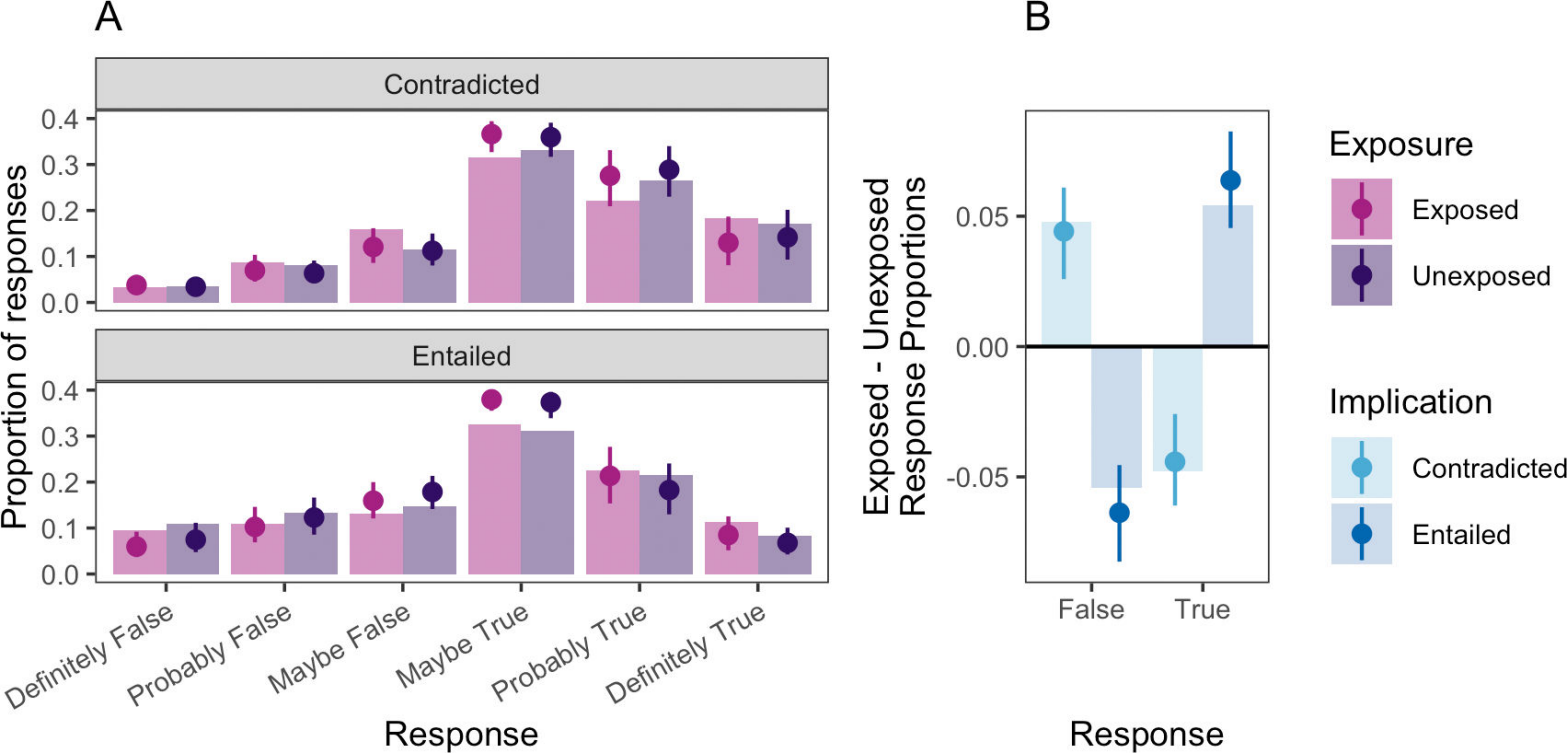
(*a*) Test-phase responses and model estimates for implication statements in experiment 1, broken down by exposure (exposed versus unexposed) and implication type (contradicted and entailed). Bars represent observed proportions. Points and error bars indicate model predictions with 95% credible intervals. *(b*) Difference in proportions of responses between exposed and unexposed conditions at test, aggregated across true and false response options. Bars indicate observed differences, with points and error bars indicating binary logistic regression model predictions and 50% credible intervals.

Figure 2 shows participants’ truth judgements for the implication statements in the quiz condition of experiment 1. Figure 2*a* shows a detailed look at the pattern of responding across experimental conditions. As shown, a larger proportion of responses for ‘maybe …’, ‘probably …’ and ‘definitely true’ and a smaller proportion of ‘maybe …’, ‘probably …’ and ‘definitely false’ responses were observed when participants were exposed to the entailing premise statements. Crucially, this pattern was reversed for the contradicted statements: fewer participants chose responses indicating truth when they were exposed to the contradicting premises and instead more chose responses indicating falsehood. Figure 2*b* shows this comparison more clearly by aggregating across the different ‘true’ and ‘false’ responses and computing a difference in proportions between statements where participants were exposed to premise statements or not. Again, contradictory premises increased false responses and decreased true responses, and the opposite pattern was observed for entailing statements.

An increase in truth ratings for the entailed statements could potentially be explained by familiarity or fluency. This would be consistent with findings from Arkes and colleagues [24], who found evidence for illusory truth in novel statements that were on the same topic as previously seen statements. However, simple metacognitive cues cannot explain the decrease in truth ratings for the contradicted items, as familiarity or fluency should encourage uniformly higher, not lower, truth ratings.

A regression model was used to tease apart the possible metacognitive effects of incidental exposure and logical implications. The model includes (i) a binary variable indicating the type of implication statement’s actual truth value (true or false), (ii) a binary predictor for prior exposure to the related premise coded 0 when the premise was not seen and 1 when it was seen (capturing potential unidirectional metacognitive effects of exposure), and (iii) a variable capturing the effect of implications, coded 1 for implied truth, −1 for implied falsehood and 0 when the related premise statement was not seen at exposure. Thus the ‘implication’ predictor accounts for the effect of exposure to the premise on truth judgements for the implied statements (either positive or negative), while the ‘exposure’ predictor accounts for any positive effect from metacognitive cues. We also incorporated ‘maximal’ random intercepts and slopes for all terms varying by subject and item [40]. Expressed in the common ‘lme4’ syntax [41], the regression model was


$$\;\begin{array}{rl} response∼\text{statement truth} & +\;exposure+\text{implication}\\ & +\left(1+\text{exposure}+\text{implication}|\text{subject}\right)\\ & +\left(1+\text{exposure}+\text{implication}|\text{item}\right). \end{array}$$

Table 1 presents a summary of the posterior distribution estimated for the population-level coefficients in this model. The results indicate that both familiarity and the implication of the premise statements affected participants’ truth ratings. Figure 1 also visualizes the relevant posterior predictive distributions for this model’s fixed effects, ignoring the random effects structure. The model does well in predicting the proportions of responses, with approximately accurate coverage as expected for the different sets of intervals. It is worth noting that these model estimates cannot be expected to perfectly correspond to the observed proportions because the effects of individual items and participants have been (at least partially) accounted for in the random effects of the models.

**Table 1.** Population coefficients and 95% credible intervals of Bayesian regression model for implication effects in experiment 1.

term	estimate	CI_2.5%_	CI_97.5%_
intercept [16]	−3.37	−3.82	−2.91
intercept [24]	−2.24	−2.69	−1.79
intercept [34]	−1.34	−1.78	−0.9
intercept [1]	0.28	−0.15	0.74
intercept [40]	1.82	1.38	2.28
exposure	0.07	−0.09	0.23
implication	0.17	0.02	0.34
statement truth	−0.83	−1.46	−0.21

Somewhat surprisingly, the effects of exposure on participants’ truth ratings for the premise statements were very subtle. Our primary preregistered test compared participants’ truth ratings for the exposed premise statements at test against their ratings for another set of eight unexposed premise statements. This analysis did not find evidence of any meaningful illusory truth effect, with a posterior estimate for the exposure parameter near zero. However, an additional preregistered analysis comparing participants’ initial truth judgements for the premise statements to their later truth judgements for those same statements at test did find an increase in endorsements, β= 0.213, 95% CI [0.086, 0.341].

Although the ‘quiz’ presentation used in experiment 1 is traditionally seen as providing ‘incidental exposure’ to statements, asking participants to judge the truth of these items may have led them to feel pressured to maintain consistency in their responses. Participants showed a general bias towards judging statements ‘true’ during the exposure phase, responding with some variation of ‘true’ for 71.8% of responses. Attempting to maintain consistency with these prior responses could have thereby influenced their responses to the implication statements, and thus the observed effect may be an artefact of the experimental context.

#### 3.2.3. Secondary and exploratory analyses

Examination of posterior predictive plots across predictor levels indicates some modest misspecification of the model. To corroborate the primary preregistered analysis, we conducted a secondary logistic regression analysis after binarizing responses into ‘true’ and ‘false’ categories. The estimate of the implication effect in this model was consistent with the primary preregistered analysis β= 0.281, 95% CI [0.076, 0.48]. This model’s predictions are plotted in figure 2*b*.

## 4. Experiment 2

Experiment 2 was conducted to address the possibility that pressure for internal consistency produced the effects on the implication-statement truth ratings observed in experiment 1.

### 4.1. Methods

#### 4.1.1. Participants

A total of 300 participants were recruited from CloudResearch using procedures identical to experiment 1. As before, participants who failed attention check questions or who indicated they had looked up answers were excluded from analyses, leaving a final sample of 286 (157 female, median age 38 years old).

#### 4.1.2. Materials and procedures

All items and procedures were identical to experiment 1 except for three changes.

First, all participants were assigned to a new ‘interest’ condition. At exposure, participants were told that they would see a set of statements, some true and some false, and they were instructed to rate how interesting each statement was. This change was made to avoid forcing participants to make a true/false judgement for the exposed premise statements, which could have pushed them to try to make coherent or consistent responses to the related implication statements. Importantly, as with a true/false quiz, the presentation of statements in this context should not warrant any inference as to the truth of those statements.

Second, to provide a longer period of distraction between exposure and test, the ordering of the items in the test phase was rearranged. Participants first judged the truth of 24 control items (12 true and 12 false) to extend the time between viewing the main test items.

Third, another change in test presentation order further prevented any consistency pressure: participants judged the truth of all of the implication statements before judging the premise statements.

### 4.2. Results

**Figure 3.**
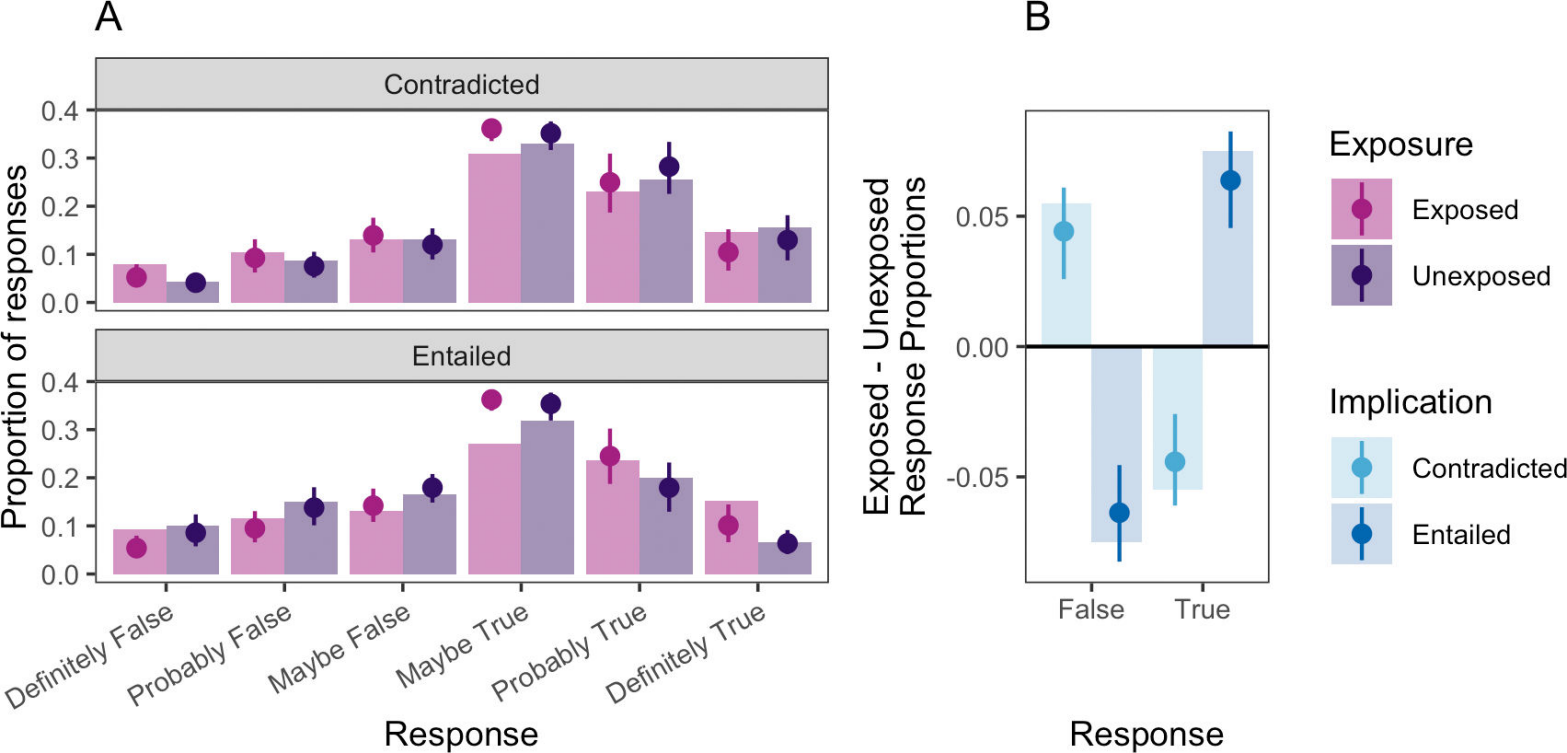
(*a*) Test-phase responses and model estimates for implication statements in experiment 2, broken down by exposure (exposed versus unexposed) and implication type (contradicted and entailed). Bars represent observed proportions. Points and error bars indicate model predictions with 95% credible intervals. *(b*) Difference in proportions of responses between exposed and unexposed conditions at test, aggregated across true and false response options. Bars indicate observed differences, with points and error bars indicating binary logistic regression model predictions and 50% credible intervals.

Figure 3 shows participants’ average truth ratings for the implication statements with and without exposure to their corresponding premise statements in experiment 2. The illusory implication effect was again observed. As shown in the figure, truth ratings were clearly affected by exposure: participants gave higher truth ratings following exposure to entailed statements, and lower truth ratings following exposure to contradicted statements. Table 2 shows the posterior population-level estimates for an identical regression as was conducted for experiment 1. Experiment 2 replicated the effects of experiment 1 in a revised design that eliminated any consistency pressure or demands on participants. In fact, the magnitude of the effect for the implication statements was somewhat larger in experiment 2 than in experiment 1.

**Table 2.** Population coefficients and 95% credible intervals of Bayesian regression model for implication effects in experiment 2.

term	estimate	CI_2.5%_	CI_97.5%_
intercept [16]	−3.17	−3.59	−2.75
intercept [24]	−2.04	−2.46	−1.63
intercept [34]	−1.18	−1.59	−0.77
intercept [1]	0.36	−0.04	0.78
intercept [40]	1.92	1.51	2.35
exposure	0.13	−0.03	0.29
implication	0.38	0.20	0.56
statement truth	−0.79	−1.37	−0.23

In addition, participants’ truth ratings for the premise statements in experiment 2 revealed the classic illusory truth effect: premise statements were rated as more true when participants had been exposed to them previously β= 0.516, 95% CI [0.319, 0.713].

The illusory implication effect for the implication statements was generally similar in magnitude to the illusory truth effect observed among the premise statements.

#### 4.2.1. Secondary and exploratory analyses

Examination of posterior predictive plots across predictor levels indicates a good fit of the ordinal regression model to participants’ observed responses. Nevertheless, for consistency of presentation, we conducted a secondary logistic regression analysis after binarizing responses into ‘true’ and ‘false’ categories. The estimate of the implication effect in this model was consistent with the primary preregistered analysis β= 0.297, 95% CI [0.062, 0.534]. This model’s predictions are plotted in figure 3*b*.

## 5. Experiment 3

Finally, we sought to replicate these findings once again and to rule out the possibility that the illusory implication effect is a product of experimental idiosyncrasies, such as misapprehension of instructions or potential task demands. In experiment 3, we ensured participants were attending carefully and understood the instructions through additional attention and comprehension checks. In addition, we evaluated the potential for unanticipated task demands by excluding from the analysis any participants who had suspicions about the study or who guessed its true purpose.

### 5.1. Method

#### 5.1.1. Participants

We recruited 540 participants from CloudResearch’s Connect service and excluded participants who failed attention or instruction comprehension checks, leaving a final sample of 475 (227 female, median age 38 years old). In a secondary set of preregistered analyses, we also excluded participants who suspected that the study had an undisclosed purpose (49) and additionally, those who guessed the true purpose of the study in an open-ended response (70), leaving 356 participants for these analyses.

#### 5.1.2. Materials and procedures

All item pairs were identical to experiment 2. However, several changes were made to the study’s procedures to limit the potential that the illusory implication effect could be produced by idiosyncratic features of the experimental task.

First, we sought to further ensure that participants would not mistake the exposure context for a communicative context, while also not inducing any consistency pressure at the test. To this end, we created a new ‘believability’ task for the exposure phase. Participants were instructed to carefully read two statements and select the one that they thought would be more believable to the average person. This task should provide a pragmatic context for incidental exposure where it was explicitly and pragmatically clear that the statements could be either true or false, but without inducing the consistency pressure that might come with a true or false ‘quiz’. To further ensure this task provided incidental exposure, participants were reminded that statements could be true or false each time they judged their believability and comprehension check questions were included before and after the exposure phase to ensure participants remembered this aspect of the task.

To improve our ability to measure the implication effect, in experiment 3 participants were exposed to 12 of the premise statements at exposure rather than eight. As a consequence, we did not examine illusory truth effects. The 24 premise statements were randomly sorted into 12 comparison questions, and each participant saw six of these pairs at exposure, along with five control pairs.

Following the test phase, two new questions assessed the potential influence of task demands. Participants were asked if they ever suspected the study had an undisclosed purpose (yes/no), and then regardless of their answer, asked to explain their suspicions in an open-ended response. Responses were coded to identify participants who guessed that the true purpose concerned how their responses to the test phase would be affected by the exposure phase or by seeing misinformation. These participants were excluded from certain analyses following our preregistered analysis plan.

Finally, six attention check questions were more tightly integrated into the task to ensure better attendant response. Following the ‘infrequency-items’ approach of [42], exposure phase attention checks consisted of a very well-known fact and one wholly unbelievable fiction. Test phase attention checks consisted of very easy trivia items that all participants should know. Participants who answered any of the six attention checks incorrectly were excluded from analyses.

### 5.2. Results

**Figure 4.**
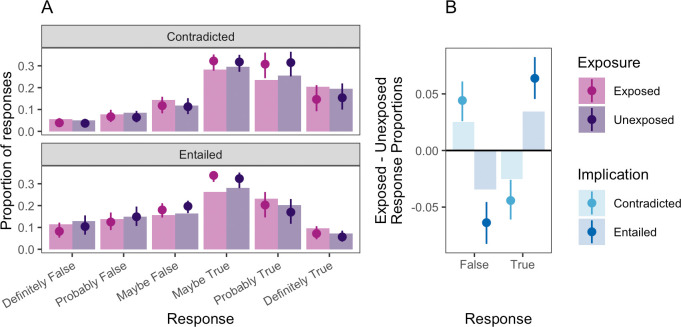
(*a*) Test-phase responses and model estimates for implication statements in experiment 3, broken down by exposure (exposed versus unexposed) and implication type (contradicted and entailed). Bars represent observed proportions. Points and error bars indicate model predictions with 95% credible intervals. (*b*) Difference in proportions of responses between exposed and unexposed conditions at test, aggregated across true and false response options. Bars indicate observed differences, with points and error bars indicating binary logistic regression model predictions and 50% credible intervals. Plotted responses and model predictions exclude participants who in any way indicated suspicion about the purpose of the study. Note that, as before, these model predictions exclude random effects of items and subjects and so will not necessarily align precisely with the observed data.

Figure 4 presents the results from experiment 3 as for prior experiments. The effects in experiment 3 are notably subtler than in prior experiments, yet the key findings were again replicated. As shown in figure 4*b*, contradictory premises increased false responses and decreased true responses, and the opposite pattern was observed for entailing statements.

#### 5.2.1. Preregistered analyses

First, in accordance with our preregistered analysis plan, we replicate the primary ‘maximal’ random effects regression analysis of experiments 1 and 2. Table 3 presents the results of this regression. The illusory implication effect was replicated, with a magnitude of the implication coefficient very similar to that observed in experiment 1.

**Table 3.** Population coefficients and 95% credible intervals of Bayesian regression model for implication effects in experiment 3.

term	estimate	CI_2.5%_	CI_97.5%_
intercept [16]	−3.31	−3.80	−2.81
intercept [24]	−2.19	−2.68	−1.71
intercept [34]	−1.35	−1.84	−0.87
intercept [1]	0.08	−0.41	0.56
intercept [40]	1.69	1.20	2.18
exposure	0.12	−0.01	0.24
implication	0.14	0.01	0.27
statement truth	−1.15	−1.84	−0.49

Next, to explore the possibility that these results might be influenced by task demands, we also carried out a parallel analysis excluding participants whose responses may have been impacted by their suspicions as to the true purpose of the study [43]. We excluded both participants who explicitly stated they were suspicious when asked directly and those who, when forced to guess in an open-ended response, guessed that the true purpose had to do with the first phase somehow affecting their responses in the second. Removing these participants did not substantially change the estimate of the implication effect, β= 0.161, 95% CI [0.018, 0.302].

#### 5.2.2. Secondary and exploratory analyses

As with experiment 1, examination of posterior predictive plots across predictor levels indicates some modest misspecification of the model. To corroborate the primary preregistered analysis, we conducted a secondary logistic regression analysis after binarizing responses into ‘true’ and ‘false’ categories. The estimate of the implication effect in this model was generally consistent with the primary preregistered analysis β= 0.136, 95% CI [−0.029, 0.297]. This model’s predictions are plotted in figure 4*b*.

**Figure 5.**
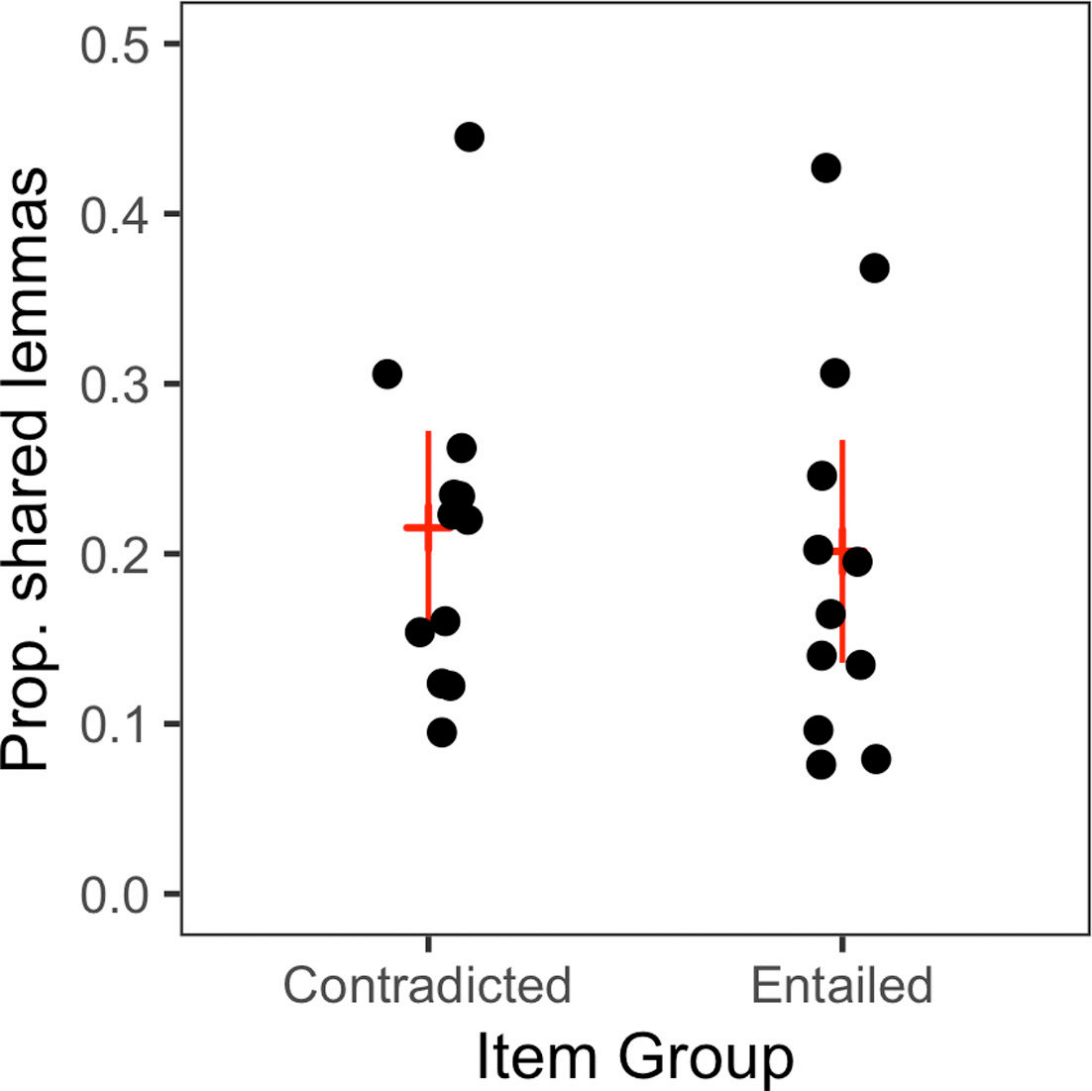
Word-level overlap between premise and implication statements (after lemmatization).

In the regression models used in experiments 1 to 3, a coefficient for prior exposure to a related statement is used to represent potential positive effects on truth ratings owing to metacognitive influences, such as fluency and familiarity. Consequently, identification of the ‘implication’ effect coefficient rests on the assumption that the directional metacognitive effects of prior exposure are not accidentally confounded with item groups (contradicted and entailed statements). To assess the potential for confounding in the familiarity or fluency that exposure to the premise statements would probably engender for the implication statements, we examined the degree of surface-level overlap within each item pair. We compared the proportion of shared words (after lemmatization) between premise and implications within each item pair. As shown in figure 5, there is no evidence for confounding between surface-level similarity and item types (median overlap = 0.19 in false-implied-true items, 0.21 in true-implied-false items) that would suggest differential effects of fluency or familiarity across types.

## 6. Discussion

We observed an ‘illusory implication’ effect across three preregistered experiments: incidental exposure to premises (e.g. that ‘The Tour de France has been held every year since its inception in 1903’) influenced participant’s truth ratings for examples of their logical implications (e.g. that ‘The Tour de France was still held during World War I and World War II’). Unlike the illusory truth effect, this effect cannot be easily explained by the effects of familiarity or fluency on the judgement process (cf. [3,8,9,24]): whereas these metacognitive cues would be expected to uniformly increase endorsements, participants’ truth ratings for implication statements were both increased and decreased according to the implications of the corresponding premises. Thus, it appears that incidental exposure has a genuine effect on people’s beliefs beyond simply creating a sense of fluency or familiarity.

What cognitive mechanisms could explain these findings? Broadly, our findings are most consistent with the perspective advocated by Gilbert *et al*. [12,44], who argued that people adopt testimony as belief by default. In line with this, a number of researchers have sought to explain how exposure to a related statement can influence truth judgements for novel statements by concluding that statements are simply believed, even following incidental exposure (e.g. [13,15]). We concur with these claims, concluding that our best explanation is that incidental exposure to the premise statements leads participants to at least partially adopt them as beliefs that then influence judgements about the implication statements via a process of inference. If so, this apparently occurs despite the participant’s awareness that the statements may be false, as was carefully ensured by constant reminders and comprehension checks in experiment 3. Simply entertaining an idea, even knowing it could be false, can apparently induce some measure of belief.

Following this same basic assumption, Unkelbach & Rom [15] have proposed a more detailed process-level account of repetition-induced truth effects. According to their ‘referential theory’, reading or hearing a statement activates the concepts to which it refers, where knowledge of these concepts is represented as a network of nodes connected by excitatory or inhibitory edges. A judgement of truth is made based on the overall coherence of the network, given the activation of concepts and their connections (e.g. an excitatory connection between two activated concepts would be coherent, but an inhibitory connection between two activated concepts would be incoherent). In a spirit similar to Gilbert [12], the theory explains how repetition can increase judgements of truth by positing that exposure to a statement modifies these links in memory: ‘If information is new … it will be instigated within memory, and the respective links will form’ [15, p. 113]. At test, repeated statements therefore have ‘on average more corresponding references which are coherently linked due to the prior presentation’ [15, p. 113].

In contrast to the referential theory, we interpret our findings by directly appealing to beliefs and inferences, rather than any more specific representation, like coherentist networks. We take beliefs and attendant inferential processes to be core elements in the basic ontology of cognitive science, and so prefer an explanation that avoids positing additional intermediary mechanisms to explain this specific finding, and that allows for a plurality of potential representations.

Nevertheless, as emphasized by the referential theory [15], interrelations among beliefs and a broader notion of ‘coherence’ are key to understanding these findings. Modern theorists generally do not conceive of beliefs as lists of propositions, but instead as subserved by structured mental models or intuitive theories of how the world works [45–49]. These mental models might be represented in a variety of formats, such as simulations (e.g. [50]); formal logics, programs or grammars (e.g. [51–53]); directed graphs (e.g. [48,54]) or, indeed, undirected graphs, such as coherentist networks (e.g. [49,55,56]). In our view, coming to believe a statement entails updating one’s relevant mental models, whatever their formats, with the new information conveyed by that statement. Importantly, coherence is a key feature of all of these mental models, such that updating them is expected to have wide-ranging and coherent implications [46,48,49]. We take the coherence of inferences following exposure to be evidence for a meaningful influence on people’s mental models, and the key to our claim that incidental exposure influences beliefs.

Finally, it is worth clarifying that our findings and wider claims about the illusory implication effect are broadly consistent with existing metacognitive explanations of the illusory truth effect: we hypothesize that the illusory implication effect is a distinct and somewhat orthogonal effect, based on appreciation of the meaning of statements rather than solely on the metacognitive experiences they produce. Nevertheless, it remains clear that fluency plays a crucial role in the classic illusory truth effect, especially as evidenced by neuroscientific findings [9] and by the findings indicating the effect can be reversed when the pragmatics of the exposure context is changed [11].

## 7. Conclusions

Whatever the cognitive mechanism behind the illusory implication effect we observed, these findings are further cause for concern about the spread and influence of misinformation. The spread of misinformation and fake news appears to outpace the spread of factual news [57] and creates massive challenges for online platforms seeking to remove, label and correct misinformation [58]. These challenges may be further compounded by apparently basic features of human cognition: that incidental exposure to false statements can lead them to be *believed*.

## Data Availability

Materials, data and analysis code for all experiments can be found at [59]. Preregistration information for experiments can be found at [60] (exp. 1), [61] (exp. 2) and [62] (exp. 3). Experiments 1 and 2 were first reported in a six-page paper presented at the *43rd Annual Meeting of the Cognitive Science Society* and appearing in the conference’s *Proceedings*. This non-archival conference paper included similar narrative motivations for the studies as presented here, with a more limited literature review. The narrative interpretations and discussion have changed in light of the findings of the novel third experiment and additional analyses reported here.

## Footnotes

^1^
These researchers used materials following those used by [34] (exp. 2), who induced incidental exposure through a series of true-or-false quizzes. In this study, contradictory statements were rated as less true following a two-week delay, but only when participants were warned that the statements had been changed. This warning provides an avenue by which metacognitive cues could be used to inform truth judgements about contradictions without inducing beliefs.
